# Crystal structure of tris­(2-di­cyclo­hexyl­phosphino-2′,6′-dimeth­oxy-1,1′-biphenyl-κ*P*)-μ-oxoethenyl­idene-*triangulo*-trigold(I) bis­(tri­fluoro­methane­sulfon­yl)imide

**DOI:** 10.1107/S2056989021003844

**Published:** 2021-04-16

**Authors:** Colin T. Hartgerink, Richard J. Staples, Carolyn E. Anderson

**Affiliations:** a Department of Chemistry and Biochemistry, Calvin University, 1726 Knollcrest Circle SE, Grand Rapids, MI 49546, USA; b Center for Crystallographic Research, Department of Chemistry, Michigan State University, 578 S. Shaw Lane, East Lansing, MI 48824, USA

**Keywords:** crystal structure, ketenyl­idene, tri-gold

## Abstract

The title ketenyl­idene, [Au_3_(C_2_O)(C_26_H_35_O_2_P)_3_](C_2_F_6_NO_4_S_2_), was obtained upon exposure of [2-(di­cyclo­hexyl­phosphino)-2′,6′-dimeth­oxy-1,1′-biphen­yl]gold(I) bis­(tri­fluoro­methane­sulfon­yl)imide to acetic anhydride at elevated temperature. The ketenyl­idene bridge caps the tri-gold cluster. The title compound has provided crystals that upon analysis represent the first tri-gold ketenyl­idene with atomic distances indicative of bonding inter­action between the gold atoms.

## Abstract 2

The title compound contains a ketenyl­idene bridge that caps a tri-gold cluster. This is the first reported tri-gold ketenyl­idene with atomic distances indicative of bonding inter­action between the gold atoms.

## Chemical context   

Metal clusters containing ketenylidenes are of inter­est for their wide range of applications. For instance, ketenylidenes are useful for facilitating C—C bond formation and cleavage (Went *et al.*, 1987 ▸), metal cluster building (Sailor & Shriver, 1985 ▸), and as potential inter­mediates for carbon monoxide chemistry (Jensen & Shriver, 1992 ▸). One of the first transition-metal ketenyl­idene complexes described was a tricobalt cluster reported by Seyferth *et al.* in 1974 (Seyferth *et al.*, 1974 ▸). Since then, the scope of ketenyl­idene clusters has been expanded to include metals such as osmium (Went *et al.*, 1987 ▸), ruthenium (Sailor & Shriver, 1985 ▸), molybdenum (Ramalakshmi *et al.*, 2015 ▸), and manganese (Crespi & Shriver, 1986 ▸) to name a few.

However, relatively few ketenylidenes involving gold have been reported. Work by Green and co-workers uncovered a surface-bound gold ketenyl­idene [Au_2_CCO], which serves as a reactive inter­mediate in the aerobic oxidation of acetic acid on Au/TiO_2_ surfaces (Green *et al.*, 2012 ▸). More recently, Daugherty and co-workers reported the first instance of a tri-gold ketenyl­idene (Daugherty *et al.*, 2017 ▸). In that case, the Au⋯Au distances suggest that there is no bonding inter­action between the metal atoms.

Herein, we describe the first crystal structure analysis of a tri-gold ketenyl­idene in which the atomic distances suggest a bonding inter­action between the gold atoms

## Structural commentary   

**Figure 1:**
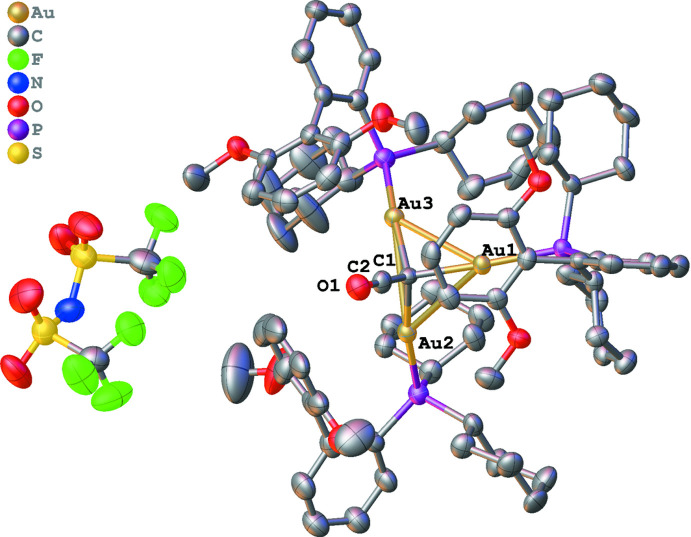
The mol­ecular structure of the title compound with select atom labeling. Displacement ellipsoids are drawn at the 50% probability level. Hydrogen atoms are omitted for clarity reasons.

The mol­ecular structure of the title compound is shown in Fig. 1 ▸. Four mol­ecules are present in the unit cell (*Z* = 4) and there is one component in the asymmetric unit. The title compound consists of three mol­ecules of (2-di­cyclo­hexyl­phosphino-2′,6′-dimeth­oxy-1,1′-biphen­yl)gold(I) bis­(tri­fluoro­methane­sulfon­yl)imide capped by a ketenyl­idene unit (C=C=O) to form a tri-gold cluster. The tri-gold cluster has an overall charge of +1, with tri­fluoro­methane­sulfonyl­imide serving as the counter-ion. As shown in Fig. 1 ▸, the ketenyl­idene atoms (C=C=O) form an angle of 88.1 (5)° with the mean Au1—Au2—Au3 gold plane.
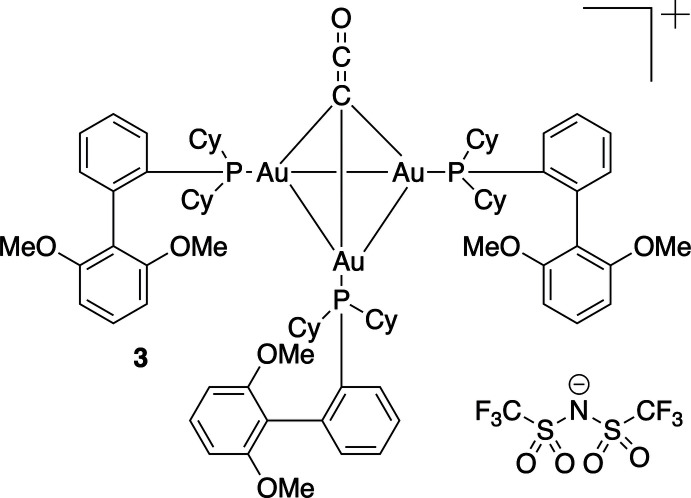


The Au—Au bond distances suggest aurophilic inter­action (Schmidbaur & Schier, 2012 ▸). The shortest Au—Au bond length is 3.1910 (5) Å (Au1-Au3), which is significantly shorter than the sum of two van der Waals radii (Bondi, 1964 ▸). The Au2—Au3 bond length of 3.2101 (5) Å also indicates a significant Au—Au inter­action, although the complex is not entirely symmetrical, with the Au1—Au2 bond length measuring 3.3005 (5) Å. Other bond lengths within the cluster are more highly conserved within each subunit of the trimeric structure [*e.g*. Au—C1 distances: 2.090 (7) to 2.098 (7) Å; Au—P distances: 2.273 (2) to 2.281 (2) Å].

## Supra­molecular features   

**Figure 2:**
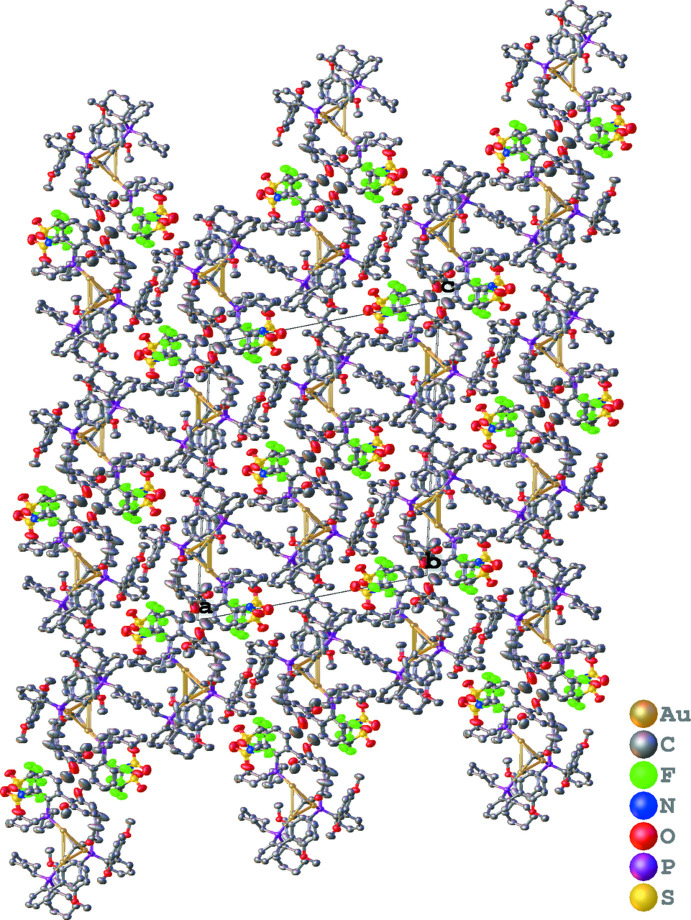
Packing diagram of the title compound along the *b* axis.

In the crystal structure of the title compound, the discrete complexes are arranged into columns along the *b* axis (Fig. 2 ▸). Within these columns, the ketenyl­idene atoms alternate between the +*a* and −*a*-axis directions. The only other similar cluster with Au was reported by Daugherty and co-workers (Daugherty *et al.*, 2017 ▸), and it does not show this alternating arrangement. However, in this earlier case, the Au atoms are all bonded to the carbon of *N*-heterocyclic carbene ligands, rather than to phosphines. As such, the title compound is the first trimetallic ketenyl­idene cluster of any metal that involves the metal bound to only phosphine and the ketenylidiene bridge, rather than the more common C=O ligand found in most trimetallic metal complexes in the CSD.

## Database survey   

The Cambridge Structural Database (CSD, Version 5.42, February 2021; Groom *et al.*, 2016 ▸) contains 12 unique structures of ketenyl­idene-bridged metal clusters. Of these, ten contain clusters of three metal atoms, including: all iron (CAXVAY, Kolis *et al.*, 1983 ▸; GEFPIQ, Bogdan *et al.*, 1988 ▸), all ruthenium (DELSAO, Sailor & Shriver, 1985 ▸; FONWAG, Sailor *et al.*, 1987 ▸), all osmium (FONYEM, Went *et al.*, 1987 ▸), and all gold(I) (LEBFOQ, Daugherty *et al.*, 2017 ▸) and various combinations of iron, cobalt, manganese, molybdenum, and ruthenium (DUHDIT, Crespi & Shriver, 1986 ▸ and Crespi *et al.*, 1988 ▸; GAHBIA, Ching *et al.*, 1988 ▸; HUQBIG, Ramalakshmi *et al.*, 2015 ▸; KALVAU, Ching *et al.*, 1989 ▸). Two additional structures of this type with central clusters of four metal atoms, either three ruthenium and one copper (PAJWOM, Gunale *et al.*, 1992 ▸) or three iron and one copper (KINFOC10, Gunale *et al.*, 1992 ▸) have also been reported. Within this group, only one 2Fe/1Co cluster bears a phosphine ligand (KALVAU, Ching *et al.*, 1989 ▸) similar to the reported title compound; however, even in this case, the reported cluster is much simpler than the title compound, as all of the remaining positions are occupied by CO.

The only other known all gold(I) cluster (LEBFOQ, Daugherty *et al.*, 2017 ▸) differs from both the reported title compound as well as the other structures in the CSD, as it bears *N*-heterocyclic carbene ligands attached to gold, rather than either phosphines or carbon monoxide ligands. Additionally, the gold(I) atoms in this previously reported cluster were too far apart from each other to have any metal–metal bonding inter­action.

Thus, the reported ketenyl­idene cluster differs from similar compounds in the CSD in both the title cluster’s unique phosphine ligands and the short Au—Au bonding inter­actions.

## Synthesis and crystallization   

**Figure 3:**
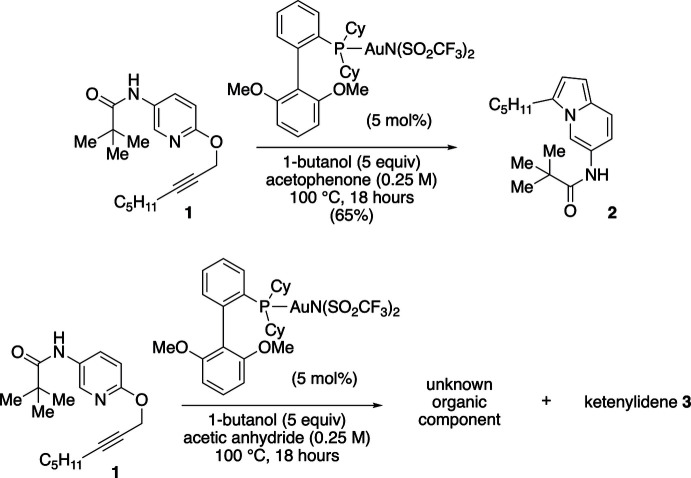
Reaction scheme. The title ketenyl­idene **3** was discovered as an unexpected by-product of a reaction exploring the gold(I)-catalyzed rearrangement of 2-propargyloxypyridine **1**.

The title compound was observed during scope studies related to the gold(I)-catalyzed synthesis of tris­ubstituted indolizine **2** from 2-propargyloxypyridine **1** (Rossler *et al.*, 2019 ▸). While initial studies had shown that treatment of pyridine **1** with methyl ketones in the presence of alcohols and (2-di­cyclo­hexyl­phosphino-2′,6′-dimeth­oxy-1,1′-biphen­yl)gold(I) bis­(tri­fluoro­methane-sulfon­yl)imide could provide tris­ubstituted indolizines **2** in moderate to good yields, when the methyl ketone was replaced with acetic anhydride, an unknown organic product and the title ketenyl­idene cluster **3** were observed (Fig. 3 ▸). In an attempt to determine the organic product of the reaction, crystals were grown by the slow evaporation of a concentrated ethanol solution over several weeks at room temperature. Using this method, a few tiny yellow needle-shaped crystals, suitable for X-ray diffraction, were obtained and analyzed. However, rather than revealing the structure of the organic product as expected, the X-ray structure revealed the title ketenyl­idene-bridged tri-gold cluster **3**. Subsequent studies aimed at the independent synthesis of cluster **3** and related species stoichiometrically were unsuccessful.

## Refinement   

Crystal data, data collection, and refinement details are collected in Table 1 ▸. All non-hydrogen atoms were refined anisotropically. Hydrogen-atom positions were calculated geometrically (C—H = 0.95–1.00 Å) and refined using a riding model with *U*_iso_(H) = 1.2*U*_eq_(C) or 1.5*U*_eq_(C-meth­yl).

## Supplementary Material

Crystal structure: contains datablock(s) I. DOI: 10.1107/S2056989021003844/yz2006sup1.cif

Structure factors: contains datablock(s) I. DOI: 10.1107/S2056989021003844/yz2006Isup2.hkl

Supporting information file. DOI: 10.1107/S2056989021003844/yz2006Isup3.cml

CCDC reference: 2070092

Additional supporting information:  crystallographic information; 3D view; checkCIF report

## Figures and Tables

**Table 1:** Experimental details

Crystal data	
Chemical formula	[Au_3_(C_2_O)(C_26_H_35_O_2_P)_3_](C_2_F_6_NO_4_S_2_)
*M*_r_	2142.59
Crystal system, space group	Monoclinic, *P*2_1_/*n*
Temperature (K)	173
*a*, *b*, *c* (Å)	24.0018 (3), 12.4867 (1), 28.4299 (3)
β (°)	103.7669 (8)
*V* (Å^3^)	8275.75 (15)
*Z*	4
Radiation type	Cu *K*α
μ (mm^−1^)	11.42
Crystal size (mm)	0.2 × 0.05 × 0.03

Data collection	
Diffractometer	Bruker APEXII CCD
Absorption correction	Multi-scan (*SADABS*; Bruker, 2013 ▸)
*T*_min_, *T*_max_	0.321, 0.735
No. of measured, independent and observed [*I* > 2σ(*I*)] reflections	73587, 15716, 11064
*R*_int_	0.110
(sin θ/λ)_max_ (Å^−1^)	0.610

Refinement	
*R*[*F* ^2^ > 2σ(*F* ^2^)], *wR*(*F* ^2^), *S*	0.048, 0.120, 1.01
No. of reflections	15716
No. of parameters	979
H-atom treatment	H-atom parameters constrained
Δρ_max_, Δρ_min_ (e Å^−3^)	1.77, −0.95

Computer programs: *COSMO* and *SAINT* (Bruker, 2013 ▸), *SHELXT* (Sheldrick, 2015*a* ▸), *SHELXL2018/3* (Sheldrick, 2015*b* ▸) and *OLEX2* (Dolomanov *et al.*, 2009 ▸).

## supplementary crystallographic information

### Crystal data

**Table:**

[Au_3_(C_2_O)(C_26_H_35_O_2_P)_3_](C_2_F_6_NO_4_S_2_)	*F*(000) = 4240
*M**_r_* = 2142.59	*D*_x_ = 1.720 Mg m^−^^3^
Monoclinic, *P*2_1_/*n*	Cu *K*α radiation, λ = 1.54178 Å
*a* = 24.0018 (3) Å	Cell parameters from 9988 reflections
*b* = 12.4867 (1) Å	θ = 2.2–70.1°
*c* = 28.4299 (3) Å	µ = 11.42 mm^−^^1^
β = 103.7669 (8)°	*T* = 173 K
*V* = 8275.75 (15) Å^3^	Needle, yellow
*Z* = 4	0.2 × 0.05 × 0.03 mm

### Data collection

**Table:**

Bruker APEXII CCD diffractometer	15716 independent reflections
Radiation source: sealed tube	11064 reflections with *I* > 2σ(*I*)
Graphite monochromator	*R*_int_ = 0.110
Detector resolution: 8.36 pixels mm^-1^	θ_max_ = 70.1°, θ_min_ = 2.2°
ω and φ scans	*h* = −29→28
Absorption correction: multi-scan (SADABS; Bruker, 2013)	*k* = −15→15
*T*_min_ = 0.321, *T*_max_ = 0.735	*l* = −34→34
73587 measured reflections	

### Refinement

**Table:**

Refinement on *F*^2^	Primary atom site location: dual
Least-squares matrix: full	Hydrogen site location: inferred from neighbouring sites
*R*[*F*^2^ > 2σ(*F*^2^)] = 0.048	H-atom parameters constrained
*wR*(*F*^2^) = 0.120	*w* = 1/[σ^2^(*F*_o_^2^) + (0.0447*P*)^2^ + 20.5744*P*] where *P* = (*F*_o_^2^ + 2*F*_c_^2^)/3
*S* = 1.01	(Δ/σ)_max_ = 0.001
15716 reflections	Δρ_max_ = 1.77 e Å^−^^3^
979 parameters	Δρ_min_ = −0.95 e Å^−^^3^
0 restraints	

### Special details

**Table:**

Experimental. Data was collected using a BRUKER CCD (charge coupled device) based diffractometer equipped with an Oxford low-temperature apparatus operating at 173 K. A suitable crystal was chosen and mounted on a nylon loop using Paratone oil. Data were measured using omega scans of 0.5° per frame for 30 s. The total number of images were based on results from the program COSMO where redundancy was expected to be 4 and completeness to 0.83Å to 100%. Cell parameters were retrieved using APEX II software and refined using SAINT on all observed reflections.Data reduction was performed using the SAINT software which corrects for Lp. Scaling and absorption corrections were applied using SADABS6 multi-scan technique, supplied by George Sheldrick. The structure was solved by the direct method using the SHELXT program and refined by least squares method on F2, SHELXL, incorporated in OLEX2.
Geometry. All esds (except the esd in the dihedral angle between two l.s. planes) are estimated using the full covariance matrix. The cell esds are taken into account individually in the estimation of esds in distances, angles and torsion angles; correlations between esds in cell parameters are only used when they are defined by crystal symmetry. An approximate (isotropic) treatment of cell esds is used for estimating esds involving l.s. planes.
Refinement. The structure was refined by Least Squares using version 2014/6 of XL (Sheldrick, 2008) incorporated in Olex2 (Dolomanov *et al.*, 2009). All non-hydrogen atoms were refined anisotropically. Hydrogen atom positions were calculated geometrically and refined using the riding model.'

### Fractional atomic coordinates and isotropic or equivalent isotropic displacement parameters (Å^2^)

**Table:**

	*x*	*y*	*z*	*U*_iso_*/*U*_eq_	
Au1	0.53937 (2)	0.36171 (3)	0.21151 (2)	0.03027 (9)	
Au2	0.54502 (2)	0.30927 (3)	0.32634 (2)	0.03393 (9)	
Au3	0.44934 (2)	0.20237 (3)	0.24086 (2)	0.03374 (9)	
P1	0.59759 (9)	0.34972 (16)	0.15898 (8)	0.0314 (4)	
P2	0.61858 (10)	0.26651 (18)	0.39059 (8)	0.0368 (5)	
P3	0.41808 (10)	0.03219 (17)	0.22248 (8)	0.0364 (5)	
O1	0.4258 (3)	0.5067 (5)	0.2643 (2)	0.0507 (16)	
O2	0.5926 (3)	0.6146 (5)	0.2136 (2)	0.0412 (14)	
O3	0.4780 (3)	0.5587 (5)	0.0590 (2)	0.0425 (14)	
O4	0.4879 (4)	0.2011 (9)	0.4500 (5)	0.118 (4)	
O5	0.5453 (4)	0.5423 (6)	0.4146 (3)	0.071 (2)	
O6	0.2659 (3)	0.1195 (5)	0.2662 (2)	0.0518 (17)	
O7	0.3411 (3)	0.2431 (6)	0.1389 (3)	0.0555 (18)	
C1	0.4875 (3)	0.3511 (6)	0.2611 (3)	0.0274 (16)	
C2	0.4552 (4)	0.4319 (7)	0.2631 (3)	0.038 (2)	
C3	0.6180 (4)	0.4679 (6)	0.1282 (3)	0.0336 (18)	
C4	0.6653 (4)	0.4587 (7)	0.1086 (3)	0.0367 (19)	
H4	0.688034	0.395750	0.115090	0.044*	
C5	0.6808 (4)	0.5367 (7)	0.0801 (3)	0.043 (2)	
H5	0.713897	0.527777	0.067595	0.052*	
C6	0.6482 (4)	0.6270 (8)	0.0698 (3)	0.043 (2)	
H6	0.657838	0.680655	0.049466	0.052*	
C7	0.6006 (4)	0.6402 (7)	0.0894 (3)	0.040 (2)	
H7	0.578703	0.704180	0.082893	0.048*	
C8	0.5845 (3)	0.5616 (6)	0.1184 (3)	0.0330 (18)	
C9	0.5331 (3)	0.5833 (6)	0.1374 (3)	0.0340 (18)	
C10	0.5377 (4)	0.6131 (6)	0.1857 (3)	0.0339 (18)	
C11	0.4892 (4)	0.6375 (7)	0.2022 (3)	0.043 (2)	
H11	0.492373	0.655634	0.235195	0.052*	
C12	0.4365 (4)	0.6351 (7)	0.1700 (4)	0.046 (2)	
H12	0.403331	0.652251	0.181156	0.055*	
C13	0.4304 (4)	0.6090 (7)	0.1224 (4)	0.044 (2)	
H13	0.393527	0.607929	0.100791	0.053*	
C14	0.4790 (4)	0.5838 (6)	0.1060 (3)	0.0366 (19)	
C15	0.6001 (5)	0.6310 (8)	0.2644 (4)	0.058 (3)	
H15A	0.584512	0.701133	0.270078	0.087*	
H15B	0.641006	0.628363	0.280373	0.087*	
H15C	0.579768	0.574788	0.277735	0.087*	
C16	0.4226 (4)	0.5417 (9)	0.0267 (4)	0.056 (3)	
H16A	0.427567	0.514674	−0.004380	0.083*	
H16B	0.401483	0.609579	0.021584	0.083*	
H16C	0.400999	0.489350	0.040980	0.083*	
C17	0.5643 (4)	0.2573 (7)	0.1096 (3)	0.0347 (18)	
H17	0.554779	0.190324	0.125365	0.042*	
C18	0.6013 (4)	0.2237 (7)	0.0751 (3)	0.044 (2)	
H18A	0.611749	0.287751	0.058530	0.053*	
H18B	0.637162	0.190220	0.093769	0.053*	
C19	0.5692 (5)	0.1446 (8)	0.0374 (4)	0.057 (3)	
H19A	0.593181	0.126978	0.014542	0.068*	
H19B	0.562425	0.077565	0.053808	0.068*	
C20	0.5121 (5)	0.1897 (9)	0.0094 (4)	0.057 (3)	
H20A	0.519205	0.251229	−0.010338	0.068*	
H20B	0.491462	0.134176	−0.013001	0.068*	
C21	0.4745 (5)	0.2260 (9)	0.0425 (4)	0.057 (3)	
H21A	0.462137	0.162899	0.058536	0.069*	
H21B	0.439727	0.261723	0.023096	0.069*	
C22	0.5074 (4)	0.3038 (7)	0.0810 (4)	0.044 (2)	
H22A	0.514978	0.370838	0.065036	0.053*	
H22B	0.483260	0.321855	0.103709	0.053*	
C23	0.6663 (4)	0.2883 (6)	0.1892 (3)	0.0347 (18)	
H23	0.687237	0.268777	0.163914	0.042*	
C24	0.6562 (4)	0.1845 (6)	0.2160 (3)	0.041 (2)	
H24A	0.636134	0.202298	0.241689	0.049*	
H24B	0.631552	0.134861	0.192923	0.049*	
C25	0.7139 (4)	0.1300 (7)	0.2387 (4)	0.046 (2)	
H25A	0.706969	0.065621	0.256844	0.056*	
H25B	0.732180	0.106649	0.212692	0.056*	
C26	0.7544 (4)	0.2061 (8)	0.2731 (3)	0.043 (2)	
H26A	0.738786	0.220874	0.301645	0.052*	
H26B	0.792276	0.171329	0.284525	0.052*	
C27	0.7619 (4)	0.3117 (8)	0.2480 (4)	0.048 (2)	
H27A	0.785333	0.361275	0.271920	0.058*	
H27B	0.782679	0.298093	0.222420	0.058*	
C28	0.7043 (4)	0.3639 (7)	0.2255 (3)	0.0383 (19)	
H28A	0.710730	0.430873	0.208902	0.046*	
H28B	0.684717	0.382670	0.251365	0.046*	
C29	0.6169 (4)	0.3013 (7)	0.4529 (3)	0.040 (2)	
C30	0.6664 (4)	0.2764 (8)	0.4899 (4)	0.051 (2)	
H30	0.696362	0.235542	0.482178	0.061*	
C31	0.6715 (5)	0.3106 (9)	0.5367 (4)	0.058 (3)	
H31	0.704642	0.293109	0.561268	0.069*	
C32	0.6277 (5)	0.3712 (9)	0.5478 (4)	0.060 (3)	
H32	0.631325	0.397685	0.579737	0.072*	
C33	0.5792 (5)	0.3923 (8)	0.5125 (4)	0.058 (3)	
H33	0.549451	0.433181	0.520603	0.069*	
C34	0.5723 (4)	0.3561 (8)	0.4655 (4)	0.049 (2)	
C35	0.5158 (4)	0.3740 (9)	0.4305 (4)	0.052 (3)	
C36	0.4742 (5)	0.2971 (11)	0.4246 (5)	0.071 (3)	
C37	0.4210 (6)	0.3079 (13)	0.3935 (6)	0.090 (5)	
H37	0.393316	0.252199	0.389193	0.108*	
C38	0.4098 (5)	0.4049 (13)	0.3686 (5)	0.082 (4)	
H38	0.373598	0.414986	0.346543	0.098*	
C39	0.4489 (5)	0.4852 (10)	0.3748 (4)	0.061 (3)	
H39	0.440122	0.551058	0.357920	0.073*	
C40	0.5020 (4)	0.4694 (10)	0.4064 (4)	0.058 (3)	
C41	0.4611 (9)	0.1637 (19)	0.4802 (8)	0.178 (11)	
H41A	0.454369	0.086915	0.474341	0.268*	
H41B	0.424264	0.200710	0.476118	0.268*	
H41C	0.484121	0.174786	0.513242	0.268*	
C42	0.5349 (7)	0.6405 (12)	0.3875 (6)	0.106 (5)	
H42A	0.531818	0.625531	0.353136	0.160*	
H42B	0.566778	0.690217	0.399398	0.160*	
H42C	0.499126	0.672768	0.391507	0.160*	
C43	0.6354 (4)	0.1216 (7)	0.3930 (4)	0.043 (2)	
H43	0.669434	0.108234	0.420426	0.052*	
C44	0.5838 (4)	0.0590 (7)	0.4024 (4)	0.045 (2)	
H44A	0.575897	0.082988	0.433312	0.054*	
H44B	0.549456	0.074185	0.376123	0.054*	
C45	0.5956 (5)	−0.0607 (8)	0.4046 (4)	0.053 (3)	
H45A	0.628158	−0.076379	0.432397	0.064*	
H45B	0.561465	−0.099116	0.409858	0.064*	
C46	0.6095 (5)	−0.1007 (7)	0.3584 (4)	0.059 (3)	
H46A	0.575521	−0.092007	0.331002	0.070*	
H46B	0.619145	−0.177892	0.361620	0.070*	
C47	0.6599 (5)	−0.0383 (7)	0.3481 (4)	0.055 (3)	
H47A	0.667039	−0.062736	0.316940	0.066*	
H47B	0.694749	−0.053823	0.373861	0.066*	
C48	0.6493 (5)	0.0829 (7)	0.3458 (4)	0.048 (2)	
H48A	0.616908	0.099917	0.318028	0.057*	
H48B	0.683828	0.120460	0.340921	0.057*	
C49	0.6807 (4)	0.3412 (7)	0.3790 (3)	0.042 (2)	
H49	0.681878	0.324040	0.344894	0.051*	
C50	0.7410 (4)	0.3146 (8)	0.4104 (4)	0.052 (2)	
H50A	0.743247	0.334052	0.444534	0.062*	
H50B	0.748318	0.236771	0.408944	0.062*	
C51	0.7866 (4)	0.3773 (9)	0.3916 (5)	0.061 (3)	
H51A	0.825119	0.361399	0.412326	0.073*	
H51B	0.786035	0.353851	0.358255	0.073*	
C52	0.7760 (4)	0.4959 (8)	0.3918 (4)	0.055 (3)	
H52A	0.804607	0.533685	0.377895	0.066*	
H52B	0.780626	0.520844	0.425589	0.066*	
C53	0.7158 (4)	0.5233 (8)	0.3625 (4)	0.056 (3)	
H53A	0.712916	0.506597	0.327995	0.067*	
H53B	0.709109	0.601050	0.365162	0.067*	
C54	0.6692 (4)	0.4610 (7)	0.3799 (4)	0.049 (2)	
H54A	0.668976	0.483412	0.413288	0.059*	
H54B	0.631127	0.477376	0.358665	0.059*	
C55	0.3468 (4)	0.0062 (7)	0.1833 (3)	0.0379 (19)	
C56	0.3376 (4)	−0.0890 (7)	0.1569 (3)	0.043 (2)	
H56	0.368843	−0.136941	0.158874	0.051*	
C57	0.2845 (4)	−0.1157 (8)	0.1280 (4)	0.048 (2)	
H57	0.279522	−0.180323	0.109843	0.058*	
C58	0.2396 (4)	−0.0482 (8)	0.1260 (4)	0.052 (3)	
H58	0.202989	−0.065319	0.106163	0.062*	
C59	0.2471 (4)	0.0462 (9)	0.1531 (4)	0.051 (2)	
H59	0.214942	0.090974	0.152273	0.061*	
C60	0.2999 (4)	0.0761 (7)	0.1808 (3)	0.038 (2)	
C61	0.3043 (4)	0.1850 (7)	0.2036 (3)	0.040 (2)	
C62	0.2853 (4)	0.2068 (7)	0.2452 (3)	0.041 (2)	
C63	0.2845 (4)	0.3103 (8)	0.2625 (4)	0.052 (2)	
H63	0.270172	0.324800	0.290286	0.063*	
C64	0.3049 (4)	0.3914 (8)	0.2387 (4)	0.050 (2)	
H64	0.305709	0.462204	0.251004	0.060*	
C65	0.3242 (4)	0.3736 (7)	0.1976 (4)	0.051 (2)	
H65	0.337491	0.431394	0.181520	0.061*	
C66	0.3239 (4)	0.2710 (7)	0.1802 (4)	0.044 (2)	
C67	0.2525 (6)	0.1374 (10)	0.3121 (4)	0.070 (3)	
H67A	0.218638	0.183715	0.307596	0.105*	
H67B	0.285073	0.172065	0.334161	0.105*	
H67C	0.244473	0.068721	0.325783	0.105*	
C68	0.3657 (5)	0.3256 (9)	0.1159 (4)	0.068 (3)	
H68A	0.376332	0.296503	0.087230	0.102*	
H68B	0.399909	0.353722	0.138478	0.102*	
H68C	0.337692	0.383396	0.106079	0.102*	
C69	0.4678 (4)	−0.0396 (7)	0.1926 (3)	0.041 (2)	
H69	0.458485	−0.117645	0.192114	0.049*	
C70	0.5302 (4)	−0.0252 (8)	0.2206 (4)	0.056 (3)	
H70A	0.534730	−0.049412	0.254429	0.067*	
H70B	0.540854	0.051437	0.221069	0.067*	
C71	0.5697 (4)	−0.0910 (8)	0.1962 (5)	0.071 (4)	
H71A	0.610169	−0.077509	0.213111	0.085*	
H71B	0.561906	−0.168218	0.199365	0.085*	
C72	0.5613 (5)	−0.0634 (8)	0.1429 (5)	0.071 (4)	
H72A	0.576272	0.009536	0.140059	0.085*	
H72B	0.583877	−0.113960	0.128011	0.085*	
C73	0.4995 (5)	−0.0681 (9)	0.1153 (5)	0.069 (4)	
H73A	0.486483	−0.143518	0.112594	0.083*	
H73B	0.496094	−0.040098	0.082154	0.083*	
C74	0.4611 (4)	−0.0027 (7)	0.1402 (3)	0.048 (2)	
H74A	0.420620	−0.010943	0.122242	0.057*	
H74B	0.471363	0.074001	0.139774	0.057*	
C75	0.4208 (5)	−0.0479 (9)	0.2776 (4)	0.057 (3)	
H75	0.462643	−0.061847	0.290921	0.069*	
C76	0.3946 (6)	−0.1572 (8)	0.2695 (4)	0.061 (3)	
H76A	0.413062	−0.196718	0.247087	0.073*	
H76B	0.353463	−0.149391	0.253436	0.073*	
C77	0.3998 (8)	−0.2226 (10)	0.3145 (5)	0.100 (5)	
H77A	0.375486	−0.287293	0.306663	0.120*	
H77B	0.440066	−0.246404	0.326290	0.120*	
C78	0.3830 (9)	−0.1643 (11)	0.3516 (6)	0.131 (8)	
H78A	0.391791	−0.208200	0.381419	0.157*	
H78B	0.340910	−0.154361	0.342031	0.157*	
C79	0.4115 (8)	−0.0526 (13)	0.3637 (5)	0.105 (6)	
H79A	0.393233	−0.013570	0.386334	0.126*	
H79B	0.452864	−0.060859	0.379026	0.126*	
C80	0.4034 (10)	0.0097 (10)	0.3158 (5)	0.127 (8)	
H80A	0.362459	0.029214	0.304421	0.152*	
H80B	0.425722	0.077068	0.322131	0.152*	
S1A	0.24408 (13)	0.2219 (2)	0.52100 (11)	0.0607 (7)	
S2A	0.24474 (12)	0.0341 (3)	0.46694 (11)	0.0639 (7)	
F1A	0.3480 (4)	0.2976 (7)	0.5371 (3)	0.112 (3)	
F2A	0.2805 (5)	0.4110 (6)	0.5073 (3)	0.110 (3)	
F3A	0.3042 (3)	0.2920 (6)	0.4621 (3)	0.086 (2)	
F4A	0.3499 (3)	−0.0314 (6)	0.4807 (3)	0.081 (2)	
F5A	0.2957 (4)	−0.0660 (7)	0.4113 (3)	0.092 (2)	
F6A	0.3264 (4)	0.0971 (6)	0.4288 (3)	0.095 (3)	
O1A	0.1904 (4)	0.2535 (8)	0.4885 (4)	0.096 (3)	
O2A	0.2535 (5)	0.2361 (7)	0.5724 (3)	0.092 (3)	
O3A	0.2090 (4)	0.0861 (8)	0.4249 (3)	0.086 (3)	
O4A	0.2313 (4)	−0.0722 (7)	0.4819 (3)	0.085 (3)	
N1A	0.2676 (4)	0.1084 (7)	0.5117 (4)	0.065 (3)	
C1A	0.2971 (6)	0.3084 (10)	0.5070 (5)	0.068 (3)	
C2A	0.3075 (6)	0.0072 (11)	0.4477 (4)	0.073 (4)	

### Atomic displacement parameters (Å^2^)

**Table:**

	*U*^11^	*U*^22^	*U*^33^	*U*^12^	*U*^13^	*U*^23^
Au1	0.03350 (16)	0.02516 (16)	0.03426 (18)	0.00089 (14)	0.01226 (13)	−0.00147 (14)
Au2	0.03692 (18)	0.03095 (17)	0.0360 (2)	0.00151 (15)	0.01285 (14)	−0.00129 (14)
Au3	0.03603 (18)	0.02896 (17)	0.0372 (2)	0.00002 (14)	0.01056 (14)	−0.00228 (14)
P1	0.0365 (10)	0.0263 (9)	0.0337 (11)	0.0014 (9)	0.0129 (9)	−0.0010 (8)
P2	0.0391 (11)	0.0341 (11)	0.0385 (12)	0.0042 (9)	0.0117 (9)	0.0010 (9)
P3	0.0416 (12)	0.0316 (10)	0.0361 (12)	−0.0012 (9)	0.0095 (9)	0.0005 (9)
O1	0.057 (4)	0.045 (4)	0.053 (4)	0.007 (3)	0.017 (3)	0.001 (3)
O2	0.045 (3)	0.041 (3)	0.037 (3)	0.000 (3)	0.006 (3)	−0.005 (3)
O3	0.043 (3)	0.045 (3)	0.038 (4)	0.001 (3)	0.005 (3)	0.001 (3)
O4	0.074 (7)	0.098 (8)	0.201 (13)	−0.002 (6)	0.070 (8)	0.033 (8)
O5	0.078 (6)	0.059 (5)	0.070 (5)	0.008 (4)	0.006 (4)	0.012 (4)
O6	0.064 (4)	0.052 (4)	0.046 (4)	−0.008 (3)	0.025 (3)	−0.006 (3)
O7	0.061 (4)	0.056 (4)	0.057 (4)	0.002 (4)	0.028 (4)	0.008 (4)
C1	0.027 (4)	0.028 (4)	0.025 (4)	−0.002 (3)	0.004 (3)	0.000 (3)
C2	0.040 (5)	0.035 (5)	0.043 (5)	−0.003 (4)	0.017 (4)	0.001 (4)
C3	0.041 (5)	0.027 (4)	0.033 (5)	−0.003 (4)	0.009 (4)	0.000 (3)
C4	0.039 (5)	0.031 (4)	0.041 (5)	0.002 (4)	0.011 (4)	−0.001 (4)
C5	0.043 (5)	0.045 (5)	0.045 (5)	−0.011 (4)	0.017 (4)	0.000 (4)
C6	0.044 (5)	0.048 (5)	0.040 (5)	−0.010 (4)	0.013 (4)	0.003 (4)
C7	0.048 (5)	0.028 (4)	0.041 (5)	−0.003 (4)	0.005 (4)	−0.006 (4)
C8	0.035 (4)	0.030 (4)	0.032 (4)	−0.004 (3)	0.005 (3)	−0.007 (3)
C9	0.033 (4)	0.028 (4)	0.043 (5)	0.005 (3)	0.013 (4)	0.005 (4)
C10	0.044 (5)	0.018 (4)	0.042 (5)	0.002 (3)	0.013 (4)	0.002 (3)
C11	0.058 (6)	0.029 (4)	0.045 (5)	0.008 (4)	0.018 (4)	0.005 (4)
C12	0.046 (5)	0.032 (4)	0.069 (7)	0.009 (4)	0.030 (5)	0.000 (4)
C13	0.034 (5)	0.038 (5)	0.059 (6)	0.003 (4)	0.006 (4)	0.001 (4)
C14	0.040 (5)	0.028 (4)	0.040 (5)	−0.002 (4)	0.006 (4)	0.001 (4)
C15	0.074 (7)	0.050 (6)	0.048 (6)	−0.006 (6)	0.009 (5)	−0.009 (5)
C16	0.051 (6)	0.062 (6)	0.050 (6)	−0.004 (5)	0.006 (5)	0.001 (5)
C17	0.044 (5)	0.029 (4)	0.035 (5)	−0.005 (4)	0.017 (4)	−0.003 (4)
C18	0.060 (6)	0.040 (5)	0.039 (5)	0.004 (4)	0.023 (4)	−0.002 (4)
C19	0.081 (8)	0.047 (6)	0.047 (6)	−0.005 (6)	0.025 (5)	−0.018 (5)
C20	0.066 (7)	0.055 (6)	0.049 (6)	−0.009 (6)	0.012 (5)	−0.013 (5)
C21	0.055 (6)	0.068 (7)	0.047 (6)	−0.004 (5)	0.010 (5)	−0.008 (5)
C22	0.037 (5)	0.044 (5)	0.052 (6)	0.000 (4)	0.011 (4)	−0.006 (4)
C23	0.037 (4)	0.027 (4)	0.044 (5)	0.000 (4)	0.018 (4)	0.002 (4)
C24	0.055 (5)	0.026 (4)	0.042 (5)	−0.006 (4)	0.011 (4)	−0.002 (4)
C25	0.045 (5)	0.041 (5)	0.055 (6)	0.011 (4)	0.016 (4)	0.004 (4)
C26	0.036 (5)	0.054 (5)	0.041 (5)	0.006 (4)	0.013 (4)	0.001 (4)
C27	0.036 (5)	0.054 (6)	0.052 (6)	0.001 (4)	0.008 (4)	0.009 (5)
C28	0.040 (5)	0.033 (4)	0.039 (5)	0.001 (4)	0.005 (4)	0.007 (4)
C29	0.045 (5)	0.046 (5)	0.031 (5)	−0.001 (4)	0.010 (4)	0.000 (4)
C30	0.056 (6)	0.053 (6)	0.046 (6)	0.010 (5)	0.019 (5)	−0.007 (5)
C31	0.067 (7)	0.060 (6)	0.048 (6)	0.007 (6)	0.018 (5)	−0.001 (5)
C32	0.075 (8)	0.063 (7)	0.045 (6)	0.009 (6)	0.017 (5)	−0.002 (5)
C33	0.065 (7)	0.057 (6)	0.056 (7)	0.016 (5)	0.024 (5)	−0.007 (5)
C34	0.058 (6)	0.039 (5)	0.054 (6)	0.006 (5)	0.024 (5)	−0.004 (5)
C35	0.051 (6)	0.068 (7)	0.041 (6)	0.018 (5)	0.019 (4)	−0.011 (5)
C36	0.050 (6)	0.074 (8)	0.095 (10)	0.003 (6)	0.031 (6)	−0.007 (7)
C37	0.057 (8)	0.099 (11)	0.121 (13)	−0.007 (8)	0.035 (8)	−0.021 (10)
C38	0.046 (7)	0.124 (12)	0.076 (9)	0.019 (8)	0.017 (6)	−0.031 (9)
C39	0.048 (6)	0.084 (8)	0.055 (7)	0.019 (6)	0.022 (5)	−0.011 (6)
C40	0.044 (6)	0.082 (8)	0.051 (6)	0.011 (6)	0.015 (5)	−0.018 (6)
C41	0.16 (2)	0.23 (3)	0.18 (2)	0.042 (19)	0.101 (19)	0.11 (2)
C42	0.099 (11)	0.094 (11)	0.114 (13)	0.007 (9)	0.001 (10)	0.042 (10)
C43	0.045 (5)	0.029 (4)	0.057 (6)	0.002 (4)	0.015 (4)	−0.001 (4)
C44	0.043 (5)	0.046 (5)	0.048 (6)	0.000 (4)	0.015 (4)	0.002 (4)
C45	0.067 (7)	0.046 (6)	0.049 (6)	−0.006 (5)	0.019 (5)	0.004 (5)
C46	0.083 (8)	0.030 (5)	0.061 (7)	0.000 (5)	0.014 (6)	0.003 (5)
C47	0.077 (7)	0.035 (5)	0.059 (7)	0.004 (5)	0.027 (6)	−0.003 (5)
C48	0.068 (7)	0.032 (5)	0.052 (6)	0.000 (5)	0.030 (5)	0.004 (4)
C49	0.048 (5)	0.040 (5)	0.043 (5)	−0.001 (4)	0.021 (4)	−0.007 (4)
C50	0.039 (5)	0.051 (6)	0.067 (7)	0.006 (5)	0.018 (5)	0.008 (5)
C51	0.037 (5)	0.064 (7)	0.081 (8)	−0.001 (5)	0.013 (5)	0.003 (6)
C52	0.047 (6)	0.055 (6)	0.066 (7)	−0.023 (5)	0.017 (5)	−0.005 (5)
C53	0.058 (6)	0.040 (5)	0.072 (7)	−0.011 (5)	0.019 (5)	−0.007 (5)
C54	0.052 (6)	0.036 (5)	0.060 (7)	−0.003 (4)	0.016 (5)	0.000 (4)
C55	0.040 (5)	0.036 (5)	0.039 (5)	−0.002 (4)	0.012 (4)	0.001 (4)
C56	0.045 (5)	0.036 (5)	0.048 (6)	−0.004 (4)	0.014 (4)	−0.001 (4)
C57	0.047 (5)	0.045 (5)	0.054 (6)	−0.006 (4)	0.016 (5)	−0.007 (4)
C58	0.050 (6)	0.062 (6)	0.045 (6)	−0.011 (5)	0.013 (5)	−0.015 (5)
C59	0.036 (5)	0.063 (6)	0.052 (6)	0.004 (5)	0.009 (4)	−0.001 (5)
C60	0.043 (5)	0.039 (5)	0.036 (5)	−0.003 (4)	0.018 (4)	0.001 (4)
C61	0.031 (4)	0.047 (5)	0.042 (5)	−0.002 (4)	0.009 (4)	−0.003 (4)
C62	0.033 (4)	0.045 (5)	0.046 (5)	−0.002 (4)	0.013 (4)	−0.001 (4)
C63	0.052 (6)	0.046 (5)	0.061 (7)	0.000 (5)	0.020 (5)	−0.002 (5)
C64	0.051 (6)	0.041 (5)	0.056 (6)	0.013 (4)	0.009 (5)	−0.005 (5)
C65	0.043 (5)	0.035 (5)	0.073 (7)	0.004 (4)	0.010 (5)	0.009 (5)
C66	0.040 (5)	0.041 (5)	0.051 (6)	−0.002 (4)	0.014 (4)	0.012 (4)
C67	0.091 (9)	0.073 (8)	0.054 (7)	−0.031 (7)	0.032 (6)	−0.002 (6)
C68	0.081 (8)	0.070 (8)	0.062 (7)	0.005 (6)	0.036 (6)	0.016 (6)
C69	0.043 (5)	0.025 (4)	0.055 (6)	0.005 (4)	0.011 (4)	−0.006 (4)
C70	0.040 (5)	0.047 (6)	0.075 (8)	0.008 (5)	0.005 (5)	0.000 (5)
C71	0.042 (6)	0.028 (5)	0.147 (13)	0.004 (4)	0.031 (7)	0.015 (6)
C72	0.083 (9)	0.036 (5)	0.111 (11)	−0.010 (6)	0.059 (8)	−0.026 (6)
C73	0.074 (8)	0.048 (6)	0.099 (10)	−0.021 (6)	0.048 (7)	−0.026 (6)
C74	0.051 (6)	0.043 (5)	0.051 (6)	0.000 (4)	0.015 (5)	−0.006 (4)
C75	0.063 (7)	0.057 (6)	0.049 (6)	−0.012 (5)	0.007 (5)	0.011 (5)
C76	0.095 (9)	0.035 (5)	0.060 (7)	−0.001 (5)	0.033 (6)	0.011 (5)
C77	0.164 (17)	0.055 (8)	0.088 (11)	0.003 (9)	0.045 (11)	0.026 (7)
C78	0.24 (2)	0.061 (9)	0.136 (15)	0.039 (12)	0.134 (17)	0.041 (9)
C79	0.173 (17)	0.110 (12)	0.054 (8)	0.010 (12)	0.068 (10)	0.011 (8)
C80	0.28 (3)	0.047 (7)	0.090 (11)	0.004 (11)	0.108 (14)	0.000 (7)
S1A	0.0655 (17)	0.0536 (15)	0.0668 (19)	0.0006 (13)	0.0234 (14)	−0.0011 (13)
S2A	0.0580 (16)	0.0746 (18)	0.0583 (17)	0.0027 (15)	0.0125 (13)	−0.0114 (15)
F1A	0.091 (6)	0.132 (7)	0.092 (6)	−0.038 (5)	−0.019 (5)	0.025 (5)
F2A	0.175 (9)	0.061 (4)	0.113 (7)	−0.006 (5)	0.070 (7)	0.000 (5)
F3A	0.100 (6)	0.092 (5)	0.071 (5)	0.003 (5)	0.032 (4)	0.013 (4)
F4A	0.075 (5)	0.094 (5)	0.070 (5)	0.024 (4)	0.008 (4)	−0.002 (4)
F5A	0.108 (6)	0.103 (6)	0.063 (5)	0.015 (5)	0.018 (4)	−0.026 (4)
F6A	0.114 (7)	0.091 (6)	0.095 (6)	−0.014 (5)	0.055 (5)	0.002 (5)
O1A	0.068 (6)	0.091 (7)	0.125 (9)	0.025 (5)	0.014 (6)	−0.010 (6)
O2A	0.144 (9)	0.073 (6)	0.073 (6)	−0.010 (6)	0.055 (6)	−0.019 (5)
O3A	0.082 (6)	0.110 (7)	0.056 (5)	0.033 (5)	−0.005 (4)	−0.008 (5)
O4A	0.115 (8)	0.065 (5)	0.086 (6)	−0.025 (5)	0.049 (6)	−0.018 (5)
N1A	0.060 (6)	0.064 (6)	0.068 (6)	−0.004 (5)	0.007 (5)	−0.014 (5)
C1A	0.075 (8)	0.067 (7)	0.060 (8)	−0.001 (7)	0.008 (6)	−0.001 (6)
C2A	0.089 (9)	0.074 (8)	0.048 (7)	0.007 (7)	0.000 (6)	0.004 (6)

### Geometric parameters (Å, º)

**Table:**

Au1—Au2	3.3005 (5)	C41—H41B	0.9800
Au1—Au3	3.1909 (5)	C41—H41C	0.9800
Au1—P1	2.280 (2)	C42—H42A	0.9800
Au1—C1	2.094 (8)	C42—H42B	0.9800
Au2—Au3	3.2101 (5)	C42—H42C	0.9800
Au2—P2	2.281 (2)	C43—H43	1.0000
Au2—C1	2.098 (7)	C43—C44	1.539 (12)
Au3—P3	2.273 (2)	C43—C48	1.536 (13)
Au3—C1	2.090 (7)	C44—H44A	0.9900
P1—C3	1.840 (8)	C44—H44B	0.9900
P1—C17	1.846 (8)	C44—C45	1.520 (13)
P1—C23	1.837 (8)	C45—H45A	0.9900
P2—C29	1.833 (9)	C45—H45B	0.9900
P2—C43	1.852 (8)	C45—C46	1.516 (14)
P2—C49	1.853 (9)	C46—H46A	0.9900
P3—C55	1.835 (9)	C46—H46B	0.9900
P3—C69	1.854 (9)	C46—C47	1.526 (14)
P3—C75	1.847 (10)	C47—H47A	0.9900
O1—C2	1.176 (10)	C47—H47B	0.9900
O2—C10	1.366 (10)	C47—C48	1.534 (12)
O2—C15	1.427 (11)	C48—H48A	0.9900
O3—C14	1.368 (10)	C48—H48B	0.9900
O3—C16	1.441 (11)	C49—H49	1.0000
O4—C36	1.397 (16)	C49—C50	1.546 (13)
O4—C41	1.278 (18)	C49—C54	1.523 (12)
O5—C40	1.360 (13)	C50—H50A	0.9900
O5—C42	1.438 (14)	C50—H50B	0.9900
O6—C62	1.376 (11)	C50—C51	1.541 (14)
O6—C67	1.432 (12)	C51—H51A	0.9900
O7—C66	1.378 (11)	C51—H51B	0.9900
O7—C68	1.422 (12)	C51—C52	1.503 (14)
C1—C2	1.282 (12)	C52—H52A	0.9900
C3—C4	1.385 (11)	C52—H52B	0.9900
C3—C8	1.409 (11)	C52—C53	1.524 (14)
C4—H4	0.9500	C53—H53A	0.9900
C4—C5	1.373 (12)	C53—H53B	0.9900
C5—H5	0.9500	C53—C54	1.537 (13)
C5—C6	1.364 (13)	C54—H54A	0.9900
C6—H6	0.9500	C54—H54B	0.9900
C6—C7	1.394 (12)	C55—C56	1.395 (12)
C7—H7	0.9500	C55—C60	1.412 (12)
C7—C8	1.394 (12)	C56—H56	0.9500
C8—C9	1.486 (11)	C56—C57	1.383 (13)
C9—C10	1.401 (12)	C57—H57	0.9500
C9—C14	1.389 (11)	C57—C58	1.359 (14)
C10—C11	1.390 (12)	C58—H58	0.9500
C11—H11	0.9500	C58—C59	1.395 (14)
C11—C12	1.374 (13)	C59—H59	0.9500
C12—H12	0.9500	C59—C60	1.375 (12)
C12—C13	1.365 (13)	C60—C61	1.499 (12)
C13—H13	0.9500	C61—C62	1.390 (12)
C13—C14	1.390 (12)	C61—C66	1.404 (12)
C15—H15A	0.9800	C62—C63	1.386 (13)
C15—H15B	0.9800	C63—H63	0.9500
C15—H15C	0.9800	C63—C64	1.372 (14)
C16—H16A	0.9800	C64—H64	0.9500
C16—H16B	0.9800	C64—C65	1.372 (14)
C16—H16C	0.9800	C65—H65	0.9500
C17—H17	1.0000	C65—C66	1.374 (13)
C17—C18	1.533 (11)	C67—H67A	0.9800
C17—C22	1.527 (12)	C67—H67B	0.9800
C18—H18A	0.9900	C67—H67C	0.9800
C18—H18B	0.9900	C68—H68A	0.9800
C18—C19	1.523 (13)	C68—H68B	0.9800
C19—H19A	0.9900	C68—H68C	0.9800
C19—H19B	0.9900	C69—H69	1.0000
C19—C20	1.522 (15)	C69—C70	1.529 (12)
C20—H20A	0.9900	C69—C74	1.530 (13)
C20—H20B	0.9900	C70—H70A	0.9900
C20—C21	1.520 (14)	C70—H70B	0.9900
C21—H21A	0.9900	C70—C71	1.539 (14)
C21—H21B	0.9900	C71—H71A	0.9900
C21—C22	1.535 (13)	C71—H71B	0.9900
C22—H22A	0.9900	C71—C72	1.520 (17)
C22—H22B	0.9900	C72—H72A	0.9900
C23—H23	1.0000	C72—H72B	0.9900
C23—C24	1.551 (11)	C72—C73	1.504 (17)
C23—C28	1.531 (11)	C73—H73A	0.9900
C24—H24A	0.9900	C73—H73B	0.9900
C24—H24B	0.9900	C73—C74	1.524 (13)
C24—C25	1.541 (12)	C74—H74A	0.9900
C25—H25A	0.9900	C74—H74B	0.9900
C25—H25B	0.9900	C75—H75	1.0000
C25—C26	1.533 (13)	C75—C76	1.497 (14)
C26—H26A	0.9900	C75—C80	1.444 (16)
C26—H26B	0.9900	C76—H76A	0.9900
C26—C27	1.529 (13)	C76—H76B	0.9900
C27—H27A	0.9900	C76—C77	1.498 (15)
C27—H27B	0.9900	C77—H77A	0.9900
C27—C28	1.524 (11)	C77—H77B	0.9900
C28—H28A	0.9900	C77—C78	1.416 (19)
C28—H28B	0.9900	C78—H78A	0.9900
C29—C30	1.421 (13)	C78—H78B	0.9900
C29—C34	1.389 (13)	C78—C79	1.56 (2)
C30—H30	0.9500	C79—H79A	0.9900
C30—C31	1.376 (14)	C79—H79B	0.9900
C31—H31	0.9500	C79—C80	1.539 (18)
C31—C32	1.391 (14)	C80—H80A	0.9900
C32—H32	0.9500	C80—H80B	0.9900
C32—C33	1.368 (15)	S1A—O1A	1.450 (9)
C33—H33	0.9500	S1A—O2A	1.436 (9)
C33—C34	1.383 (14)	S1A—N1A	1.570 (10)
C34—C35	1.496 (14)	S1A—C1A	1.784 (13)
C35—C36	1.366 (16)	S2A—O3A	1.448 (8)
C35—C40	1.376 (16)	S2A—O4A	1.453 (9)
C36—C37	1.376 (18)	S2A—N1A	1.566 (9)
C37—H37	0.9500	S2A—C2A	1.754 (14)
C37—C38	1.40 (2)	F1A—C1A	1.321 (14)
C38—H38	0.9500	F2A—C1A	1.342 (14)
C38—C39	1.356 (17)	F3A—C1A	1.346 (14)
C39—H39	0.9500	F4A—C2A	1.301 (13)
C39—C40	1.386 (14)	F5A—C2A	1.360 (14)
C41—H41A	0.9800	F6A—C2A	1.368 (14)
			
Au3—Au1—Au2	59.247 (11)	P2—C43—H43	108.8
P1—Au1—Au2	138.37 (5)	C44—C43—P2	109.0 (6)
P1—Au1—Au3	133.55 (5)	C44—C43—H43	108.8
C1—Au1—Au2	38.1 (2)	C48—C43—P2	111.4 (6)
C1—Au1—Au3	40.3 (2)	C48—C43—H43	108.8
C1—Au1—P1	172.5 (2)	C48—C43—C44	110.0 (8)
Au3—Au2—Au1	58.675 (11)	C43—C44—H44A	109.4
P2—Au2—Au1	132.86 (6)	C43—C44—H44B	109.4
P2—Au2—Au3	141.89 (6)	H44A—C44—H44B	108.0
C1—Au2—Au1	38.0 (2)	C45—C44—C43	111.0 (8)
C1—Au2—Au3	39.9 (2)	C45—C44—H44A	109.4
C1—Au2—P2	170.9 (2)	C45—C44—H44B	109.4
Au1—Au3—Au2	62.077 (11)	C44—C45—H45A	109.4
P3—Au3—Au1	136.88 (6)	C44—C45—H45B	109.4
P3—Au3—Au2	134.46 (6)	H45A—C45—H45B	108.0
C1—Au3—Au1	40.3 (2)	C46—C45—C44	111.4 (8)
C1—Au3—Au2	40.1 (2)	C46—C45—H45A	109.4
C1—Au3—P3	173.3 (2)	C46—C45—H45B	109.4
C3—P1—Au1	122.2 (3)	C45—C46—H46A	109.6
C3—P1—C17	104.8 (4)	C45—C46—H46B	109.6
C17—P1—Au1	108.5 (3)	C45—C46—C47	110.2 (9)
C23—P1—Au1	110.1 (3)	H46A—C46—H46B	108.1
C23—P1—C3	104.3 (4)	C47—C46—H46A	109.6
C23—P1—C17	105.8 (4)	C47—C46—H46B	109.6
C29—P2—Au2	121.8 (3)	C46—C47—H47A	109.1
C29—P2—C43	104.5 (4)	C46—C47—H47B	109.1
C29—P2—C49	104.8 (4)	C46—C47—C48	112.4 (9)
C43—P2—Au2	112.2 (3)	H47A—C47—H47B	107.9
C43—P2—C49	108.7 (4)	C48—C47—H47A	109.1
C49—P2—Au2	104.0 (3)	C48—C47—H47B	109.1
C55—P3—Au3	121.0 (3)	C43—C48—H48A	109.7
C55—P3—C69	104.2 (4)	C43—C48—H48B	109.7
C55—P3—C75	105.0 (4)	C47—C48—C43	109.9 (8)
C69—P3—Au3	110.1 (3)	C47—C48—H48A	109.7
C75—P3—Au3	111.6 (4)	C47—C48—H48B	109.7
C75—P3—C69	103.4 (5)	H48A—C48—H48B	108.2
C10—O2—C15	117.5 (7)	P2—C49—H49	105.9
C14—O3—C16	117.1 (7)	C50—C49—P2	118.2 (7)
C41—O4—C36	124.5 (14)	C50—C49—H49	105.9
C40—O5—C42	116.7 (10)	C54—C49—P2	109.6 (6)
C62—O6—C67	116.4 (8)	C54—C49—H49	105.9
C66—O7—C68	116.7 (8)	C54—C49—C50	110.5 (8)
Au1—C1—Au2	103.9 (3)	C49—C50—H50A	109.7
Au3—C1—Au1	99.4 (3)	C49—C50—H50B	109.7
Au3—C1—Au2	100.1 (3)	H50A—C50—H50B	108.2
C2—C1—Au1	116.3 (6)	C51—C50—C49	109.7 (8)
C2—C1—Au2	115.6 (6)	C51—C50—H50A	109.7
C2—C1—Au3	118.8 (6)	C51—C50—H50B	109.7
O1—C2—C1	178.9 (10)	C50—C51—H51A	109.4
C4—C3—P1	117.5 (6)	C50—C51—H51B	109.4
C4—C3—C8	118.2 (8)	H51A—C51—H51B	108.0
C8—C3—P1	123.8 (6)	C52—C51—C50	111.4 (9)
C3—C4—H4	118.6	C52—C51—H51A	109.4
C5—C4—C3	122.9 (8)	C52—C51—H51B	109.4
C5—C4—H4	118.6	C51—C52—H52A	109.4
C4—C5—H5	120.4	C51—C52—H52B	109.4
C6—C5—C4	119.3 (9)	C51—C52—C53	111.0 (8)
C6—C5—H5	120.4	H52A—C52—H52B	108.0
C5—C6—H6	120.1	C53—C52—H52A	109.4
C5—C6—C7	119.7 (9)	C53—C52—H52B	109.4
C7—C6—H6	120.1	C52—C53—H53A	109.2
C6—C7—H7	119.3	C52—C53—H53B	109.2
C8—C7—C6	121.4 (8)	C52—C53—C54	112.2 (9)
C8—C7—H7	119.3	H53A—C53—H53B	107.9
C3—C8—C9	124.3 (8)	C54—C53—H53A	109.2
C7—C8—C3	118.5 (8)	C54—C53—H53B	109.2
C7—C8—C9	117.2 (7)	C49—C54—C53	110.1 (8)
C10—C9—C8	121.7 (8)	C49—C54—H54A	109.6
C14—C9—C8	119.9 (8)	C49—C54—H54B	109.6
C14—C9—C10	118.2 (8)	C53—C54—H54A	109.6
O2—C10—C9	114.5 (7)	C53—C54—H54B	109.6
O2—C10—C11	124.8 (8)	H54A—C54—H54B	108.2
C11—C10—C9	120.7 (8)	C56—C55—P3	118.8 (7)
C10—C11—H11	120.6	C56—C55—C60	118.6 (8)
C12—C11—C10	118.9 (9)	C60—C55—P3	122.5 (7)
C12—C11—H11	120.6	C55—C56—H56	119.0
C11—C12—H12	119.0	C57—C56—C55	122.0 (9)
C13—C12—C11	122.1 (9)	C57—C56—H56	119.0
C13—C12—H12	119.0	C56—C57—H57	120.5
C12—C13—H13	120.5	C58—C57—C56	119.0 (9)
C12—C13—C14	119.0 (9)	C58—C57—H57	120.5
C14—C13—H13	120.5	C57—C58—H58	119.9
O3—C14—C9	115.0 (8)	C57—C58—C59	120.3 (9)
O3—C14—C13	123.9 (8)	C59—C58—H58	119.9
C9—C14—C13	121.1 (8)	C58—C59—H59	119.1
O2—C15—H15A	109.5	C60—C59—C58	121.7 (9)
O2—C15—H15B	109.5	C60—C59—H59	119.1
O2—C15—H15C	109.5	C55—C60—C61	124.5 (8)
H15A—C15—H15B	109.5	C59—C60—C55	118.3 (9)
H15A—C15—H15C	109.5	C59—C60—C61	117.0 (8)
H15B—C15—H15C	109.5	C62—C61—C60	122.8 (8)
O3—C16—H16A	109.5	C62—C61—C66	118.0 (9)
O3—C16—H16B	109.5	C66—C61—C60	119.0 (8)
O3—C16—H16C	109.5	O6—C62—C61	115.3 (8)
H16A—C16—H16B	109.5	O6—C62—C63	123.3 (8)
H16A—C16—H16C	109.5	C63—C62—C61	121.4 (9)
H16B—C16—H16C	109.5	C62—C63—H63	120.8
P1—C17—H17	106.6	C64—C63—C62	118.4 (10)
C18—C17—P1	116.5 (6)	C64—C63—H63	120.8
C18—C17—H17	106.6	C63—C64—H64	118.9
C22—C17—P1	109.7 (6)	C65—C64—C63	122.2 (9)
C22—C17—H17	106.6	C65—C64—H64	118.9
C22—C17—C18	110.1 (7)	C64—C65—H65	120.5
C17—C18—H18A	109.5	C64—C65—C66	119.0 (9)
C17—C18—H18B	109.5	C66—C65—H65	120.5
H18A—C18—H18B	108.1	O7—C66—C61	114.4 (8)
C19—C18—C17	110.8 (8)	C65—C66—O7	124.5 (9)
C19—C18—H18A	109.5	C65—C66—C61	121.0 (9)
C19—C18—H18B	109.5	O6—C67—H67A	109.5
C18—C19—H19A	109.3	O6—C67—H67B	109.5
C18—C19—H19B	109.3	O6—C67—H67C	109.5
H19A—C19—H19B	107.9	H67A—C67—H67B	109.5
C20—C19—C18	111.7 (8)	H67A—C67—H67C	109.5
C20—C19—H19A	109.3	H67B—C67—H67C	109.5
C20—C19—H19B	109.3	O7—C68—H68A	109.5
C19—C20—H20A	109.1	O7—C68—H68B	109.5
C19—C20—H20B	109.1	O7—C68—H68C	109.5
H20A—C20—H20B	107.9	H68A—C68—H68B	109.5
C21—C20—C19	112.3 (9)	H68A—C68—H68C	109.5
C21—C20—H20A	109.1	H68B—C68—H68C	109.5
C21—C20—H20B	109.1	P3—C69—H69	108.0
C20—C21—H21A	109.6	C70—C69—P3	111.2 (7)
C20—C21—H21B	109.6	C70—C69—H69	108.0
C20—C21—C22	110.5 (8)	C70—C69—C74	109.6 (8)
H21A—C21—H21B	108.1	C74—C69—P3	111.8 (6)
C22—C21—H21A	109.6	C74—C69—H69	108.0
C22—C21—H21B	109.6	C69—C70—H70A	109.8
C17—C22—C21	112.5 (8)	C69—C70—H70B	109.8
C17—C22—H22A	109.1	C69—C70—C71	109.6 (9)
C17—C22—H22B	109.1	H70A—C70—H70B	108.2
C21—C22—H22A	109.1	C71—C70—H70A	109.8
C21—C22—H22B	109.1	C71—C70—H70B	109.8
H22A—C22—H22B	107.8	C70—C71—H71A	109.2
P1—C23—H23	108.3	C70—C71—H71B	109.2
C24—C23—P1	110.5 (6)	H71A—C71—H71B	107.9
C24—C23—H23	108.3	C72—C71—C70	112.2 (9)
C28—C23—P1	112.5 (5)	C72—C71—H71A	109.2
C28—C23—H23	108.3	C72—C71—H71B	109.2
C28—C23—C24	108.8 (7)	C71—C72—H72A	109.0
C23—C24—H24A	109.6	C71—C72—H72B	109.0
C23—C24—H24B	109.6	H72A—C72—H72B	107.8
H24A—C24—H24B	108.1	C73—C72—C71	113.1 (10)
C25—C24—C23	110.1 (7)	C73—C72—H72A	109.0
C25—C24—H24A	109.6	C73—C72—H72B	109.0
C25—C24—H24B	109.6	C72—C73—H73A	109.3
C24—C25—H25A	109.4	C72—C73—H73B	109.3
C24—C25—H25B	109.4	C72—C73—C74	111.5 (9)
H25A—C25—H25B	108.0	H73A—C73—H73B	108.0
C26—C25—C24	111.3 (7)	C74—C73—H73A	109.3
C26—C25—H25A	109.4	C74—C73—H73B	109.3
C26—C25—H25B	109.4	C69—C74—H74A	109.5
C25—C26—H26A	109.3	C69—C74—H74B	109.5
C25—C26—H26B	109.3	C73—C74—C69	110.8 (9)
H26A—C26—H26B	108.0	C73—C74—H74A	109.5
C27—C26—C25	111.4 (8)	C73—C74—H74B	109.5
C27—C26—H26A	109.3	H74A—C74—H74B	108.1
C27—C26—H26B	109.3	P3—C75—H75	104.2
C26—C27—H27A	109.3	C76—C75—P3	115.7 (8)
C26—C27—H27B	109.3	C76—C75—H75	104.2
H27A—C27—H27B	107.9	C80—C75—P3	114.2 (9)
C28—C27—C26	111.7 (7)	C80—C75—H75	104.2
C28—C27—H27A	109.3	C80—C75—C76	112.5 (11)
C28—C27—H27B	109.3	C75—C76—H76A	108.6
C23—C28—H28A	109.5	C75—C76—H76B	108.6
C23—C28—H28B	109.5	C75—C76—C77	114.8 (11)
C27—C28—C23	110.7 (7)	H76A—C76—H76B	107.5
C27—C28—H28A	109.5	C77—C76—H76A	108.6
C27—C28—H28B	109.5	C77—C76—H76B	108.6
H28A—C28—H28B	108.1	C76—C77—H77A	109.2
C30—C29—P2	117.2 (7)	C76—C77—H77B	109.2
C34—C29—P2	124.2 (7)	H77A—C77—H77B	107.9
C34—C29—C30	118.4 (9)	C78—C77—C76	112.0 (12)
C29—C30—H30	119.5	C78—C77—H77A	109.2
C31—C30—C29	121.1 (10)	C78—C77—H77B	109.2
C31—C30—H30	119.5	C77—C78—H78A	108.3
C30—C31—H31	120.4	C77—C78—H78B	108.3
C30—C31—C32	119.3 (11)	C77—C78—C79	115.9 (13)
C32—C31—H31	120.4	H78A—C78—H78B	107.4
C31—C32—H32	120.2	C79—C78—H78A	108.3
C33—C32—C31	119.7 (10)	C79—C78—H78B	108.3
C33—C32—H32	120.2	C78—C79—H79A	110.2
C32—C33—H33	118.9	C78—C79—H79B	110.2
C32—C33—C34	122.1 (10)	H79A—C79—H79B	108.5
C34—C33—H33	118.9	C80—C79—C78	107.5 (13)
C29—C34—C35	122.3 (9)	C80—C79—H79A	110.2
C33—C34—C29	119.2 (10)	C80—C79—H79B	110.2
C33—C34—C35	118.5 (9)	C75—C80—C79	114.5 (12)
C36—C35—C34	119.7 (11)	C75—C80—H80A	108.6
C36—C35—C40	117.7 (11)	C75—C80—H80B	108.6
C40—C35—C34	122.3 (10)	C79—C80—H80A	108.6
C35—C36—O4	117.6 (12)	C79—C80—H80B	108.6
C35—C36—C37	123.3 (14)	H80A—C80—H80B	107.6
C37—C36—O4	119.0 (13)	O1A—S1A—N1A	116.1 (6)
C36—C37—H37	121.7	O1A—S1A—C1A	105.4 (6)
C36—C37—C38	116.7 (14)	O2A—S1A—O1A	121.1 (6)
C38—C37—H37	121.7	O2A—S1A—N1A	107.9 (6)
C37—C38—H38	119.0	O2A—S1A—C1A	101.7 (6)
C39—C38—C37	122.0 (13)	N1A—S1A—C1A	101.8 (6)
C39—C38—H38	119.0	O3A—S2A—O4A	121.5 (6)
C38—C39—H39	120.6	O3A—S2A—N1A	115.4 (6)
C38—C39—C40	118.7 (13)	O3A—S2A—C2A	102.3 (6)
C40—C39—H39	120.6	O4A—S2A—N1A	111.2 (5)
O5—C40—C35	114.2 (10)	O4A—S2A—C2A	100.6 (6)
O5—C40—C39	124.3 (12)	N1A—S2A—C2A	101.8 (6)
C35—C40—C39	121.4 (12)	S2A—N1A—S1A	126.9 (7)
O4—C41—H41A	109.5	F1A—C1A—S1A	113.1 (9)
O4—C41—H41B	109.5	F1A—C1A—F2A	108.9 (11)
O4—C41—H41C	109.5	F1A—C1A—F3A	106.9 (11)
H41A—C41—H41B	109.5	F2A—C1A—S1A	110.4 (9)
H41A—C41—H41C	109.5	F2A—C1A—F3A	104.9 (10)
H41B—C41—H41C	109.5	F3A—C1A—S1A	112.2 (9)
O5—C42—H42A	109.5	F4A—C2A—S2A	115.4 (9)
O5—C42—H42B	109.5	F4A—C2A—F5A	106.6 (11)
O5—C42—H42C	109.5	F4A—C2A—F6A	108.2 (12)
H42A—C42—H42B	109.5	F5A—C2A—S2A	109.1 (9)
H42A—C42—H42C	109.5	F5A—C2A—F6A	106.3 (10)
H42B—C42—H42C	109.5	F6A—C2A—S2A	110.8 (9)
			
Au1—P1—C3—C4	161.8 (5)	C34—C35—C40—O5	4.4 (14)
Au1—P1—C3—C8	−26.2 (8)	C34—C35—C40—C39	−178.6 (9)
Au1—P1—C17—C18	−170.1 (6)	C35—C36—C37—C38	−2 (2)
Au1—P1—C17—C22	63.9 (6)	C36—C35—C40—O5	179.2 (10)
Au1—P1—C23—C24	48.8 (6)	C36—C35—C40—C39	−3.8 (16)
Au1—P1—C23—C28	−73.0 (6)	C36—C37—C38—C39	−1 (2)
Au2—P2—C29—C30	−176.5 (6)	C37—C38—C39—C40	1.1 (18)
Au2—P2—C29—C34	−0.7 (10)	C38—C39—C40—O5	177.9 (10)
Au2—P2—C43—C44	−65.2 (7)	C38—C39—C40—C35	1.2 (16)
Au2—P2—C43—C48	56.4 (7)	C40—C35—C36—O4	−178.5 (11)
Au2—P2—C49—C50	−168.7 (7)	C40—C35—C36—C37	4.4 (18)
Au2—P2—C49—C54	63.7 (7)	C41—O4—C36—C35	122 (2)
Au3—P3—C55—C56	153.7 (6)	C41—O4—C36—C37	−61 (3)
Au3—P3—C55—C60	−29.8 (9)	C42—O5—C40—C35	175.4 (11)
Au3—P3—C69—C70	48.3 (7)	C42—O5—C40—C39	−1.5 (17)
Au3—P3—C69—C74	−74.5 (7)	C43—P2—C29—C30	55.1 (8)
Au3—P3—C75—C76	173.0 (8)	C43—P2—C29—C34	−129.1 (8)
Au3—P3—C75—C80	39.9 (13)	C43—P2—C49—C50	−48.9 (8)
P1—C3—C4—C5	172.4 (7)	C43—P2—C49—C54	−176.6 (7)
P1—C3—C8—C7	−171.8 (6)	C43—C44—C45—C46	−57.7 (12)
P1—C3—C8—C9	8.8 (12)	C44—C43—C48—C47	−56.1 (11)
P1—C17—C18—C19	178.0 (7)	C44—C45—C46—C47	55.9 (12)
P1—C17—C22—C21	−174.4 (7)	C45—C46—C47—C48	−55.7 (12)
P1—C23—C24—C25	176.7 (6)	C46—C47—C48—C43	56.2 (12)
P1—C23—C28—C27	−177.4 (6)	C48—C43—C44—C45	57.5 (10)
P2—C29—C30—C31	172.5 (8)	C49—P2—C29—C30	−59.2 (8)
P2—C29—C34—C33	−170.3 (8)	C49—P2—C29—C34	116.6 (9)
P2—C29—C34—C35	12.0 (14)	C49—P2—C43—C44	−179.7 (6)
P2—C43—C44—C45	180.0 (7)	C49—P2—C43—C48	−58.1 (8)
P2—C43—C48—C47	−177.1 (7)	C49—C50—C51—C52	57.7 (12)
P2—C49—C50—C51	174.5 (7)	C50—C49—C54—C53	57.0 (11)
P2—C49—C54—C53	−171.1 (7)	C50—C51—C52—C53	−55.9 (13)
P3—C55—C56—C57	177.7 (7)	C51—C52—C53—C54	55.0 (12)
P3—C55—C60—C59	−175.4 (7)	C52—C53—C54—C49	−55.5 (11)
P3—C55—C60—C61	9.0 (12)	C54—C49—C50—C51	−58.2 (11)
P3—C69—C70—C71	176.8 (7)	C55—P3—C69—C70	179.4 (7)
P3—C69—C74—C73	−176.1 (7)	C55—P3—C69—C74	56.6 (7)
P3—C75—C76—C77	177.2 (10)	C55—P3—C75—C76	40.3 (10)
P3—C75—C80—C79	−174.0 (12)	C55—P3—C75—C80	−92.8 (12)
O2—C10—C11—C12	178.6 (8)	C55—C56—C57—C58	−1.3 (15)
O4—C36—C37—C38	−179.3 (12)	C55—C60—C61—C62	−105.9 (11)
O6—C62—C63—C64	180.0 (9)	C55—C60—C61—C66	79.5 (12)
C3—P1—C17—C18	57.9 (7)	C56—C55—C60—C59	1.1 (13)
C3—P1—C17—C22	−68.1 (7)	C56—C55—C60—C61	−174.5 (8)
C3—P1—C23—C24	−178.5 (6)	C56—C57—C58—C59	−0.4 (15)
C3—P1—C23—C28	59.7 (7)	C57—C58—C59—C60	2.6 (16)
C3—C4—C5—C6	−0.8 (14)	C58—C59—C60—C55	−2.9 (15)
C3—C8—C9—C10	75.4 (11)	C58—C59—C60—C61	173.0 (9)
C3—C8—C9—C14	−110.5 (10)	C59—C60—C61—C62	78.5 (12)
C4—C3—C8—C7	0.2 (12)	C59—C60—C61—C66	−96.1 (10)
C4—C3—C8—C9	−179.2 (8)	C60—C55—C56—C57	1.0 (14)
C4—C5—C6—C7	1.7 (14)	C60—C61—C62—O6	4.7 (13)
C5—C6—C7—C8	−1.7 (13)	C60—C61—C62—C63	−173.3 (9)
C6—C7—C8—C3	0.7 (12)	C60—C61—C66—O7	−4.1 (12)
C6—C7—C8—C9	−179.8 (8)	C60—C61—C66—C65	174.6 (9)
C7—C8—C9—C10	−104.1 (9)	C61—C62—C63—C64	−2.2 (15)
C7—C8—C9—C14	70.1 (10)	C62—C61—C66—O7	−178.9 (8)
C8—C3—C4—C5	−0.2 (13)	C62—C61—C66—C65	−0.3 (14)
C8—C9—C10—O2	−3.3 (11)	C62—C63—C64—C65	2.1 (15)
C8—C9—C10—C11	177.1 (7)	C63—C64—C65—C66	−1.1 (15)
C8—C9—C14—O3	2.8 (11)	C64—C65—C66—O7	178.6 (9)
C8—C9—C14—C13	−176.9 (8)	C64—C65—C66—C61	0.1 (14)
C9—C10—C11—C12	−1.9 (12)	C66—C61—C62—O6	179.3 (8)
C10—C9—C14—O3	177.1 (7)	C66—C61—C62—C63	1.3 (14)
C10—C9—C14—C13	−2.5 (12)	C67—O6—C62—C61	172.9 (9)
C10—C11—C12—C13	0.3 (14)	C67—O6—C62—C63	−9.2 (14)
C11—C12—C13—C14	0.1 (14)	C68—O7—C66—C61	−175.1 (9)
C12—C13—C14—O3	−178.5 (8)	C68—O7—C66—C65	6.3 (14)
C12—C13—C14—C9	1.1 (13)	C69—P3—C55—C56	29.3 (8)
C14—C9—C10—O2	−177.5 (7)	C69—P3—C55—C60	−154.2 (7)
C14—C9—C10—C11	2.9 (12)	C69—P3—C75—C76	−68.7 (10)
C15—O2—C10—C9	−172.4 (7)	C69—P3—C75—C80	158.2 (12)
C15—O2—C10—C11	7.2 (12)	C69—C70—C71—C72	55.0 (12)
C16—O3—C14—C9	170.5 (8)	C70—C69—C74—C73	60.1 (10)
C16—O3—C14—C13	−9.8 (12)	C70—C71—C72—C73	−51.6 (12)
C17—P1—C3—C4	−74.6 (7)	C71—C72—C73—C74	51.4 (12)
C17—P1—C3—C8	97.4 (7)	C72—C73—C74—C69	−55.8 (12)
C17—P1—C23—C24	−68.2 (7)	C74—C69—C70—C71	−59.1 (10)
C17—P1—C23—C28	170.0 (6)	C75—P3—C55—C56	−79.1 (8)
C17—C18—C19—C20	56.0 (11)	C75—P3—C55—C60	97.4 (8)
C18—C17—C22—C21	56.1 (10)	C75—P3—C69—C70	−71.0 (8)
C18—C19—C20—C21	−54.9 (12)	C75—P3—C69—C74	166.2 (7)
C19—C20—C21—C22	53.1 (12)	C75—C76—C77—C78	49 (2)
C20—C21—C22—C17	−54.4 (12)	C76—C75—C80—C79	51 (2)
C22—C17—C18—C19	−56.2 (10)	C76—C77—C78—C79	−51 (2)
C23—P1—C3—C4	36.4 (8)	C77—C78—C79—C80	52 (2)
C23—P1—C3—C8	−151.5 (7)	C78—C79—C80—C75	−51 (2)
C23—P1—C17—C18	−52.0 (7)	C80—C75—C76—C77	−48.9 (17)
C23—P1—C17—C22	−178.0 (6)	O1A—S1A—N1A—S2A	10.1 (11)
C23—C24—C25—C26	56.7 (10)	O1A—S1A—C1A—F1A	173.3 (10)
C24—C23—C28—C27	59.8 (9)	O1A—S1A—C1A—F2A	51.0 (10)
C24—C25—C26—C27	−53.5 (10)	O1A—S1A—C1A—F3A	−65.6 (10)
C25—C26—C27—C28	53.8 (11)	O2A—S1A—N1A—S2A	149.6 (8)
C26—C27—C28—C23	−57.6 (10)	O2A—S1A—C1A—F1A	46.2 (11)
C28—C23—C24—C25	−59.4 (9)	O2A—S1A—C1A—F2A	−76.1 (10)
C29—P2—C43—C44	68.8 (7)	O2A—S1A—C1A—F3A	167.3 (9)
C29—P2—C43—C48	−169.6 (7)	O3A—S2A—N1A—S1A	17.2 (10)
C29—P2—C49—C50	62.4 (8)	O3A—S2A—C2A—F4A	173.8 (10)
C29—P2—C49—C54	−65.3 (8)	O3A—S2A—C2A—F5A	−66.3 (10)
C29—C30—C31—C32	−0.4 (16)	O3A—S2A—C2A—F6A	50.4 (10)
C29—C34—C35—C36	85.6 (13)	O4A—S2A—N1A—S1A	−126.4 (8)
C29—C34—C35—C40	−99.8 (12)	O4A—S2A—C2A—F4A	−60.4 (11)
C30—C29—C34—C33	5.4 (15)	O4A—S2A—C2A—F5A	59.5 (9)
C30—C29—C34—C35	−172.3 (10)	O4A—S2A—C2A—F6A	176.2 (8)
C30—C31—C32—C33	2.5 (17)	N1A—S1A—C1A—F1A	−65.1 (11)
C31—C32—C33—C34	−0.5 (18)	N1A—S1A—C1A—F2A	172.6 (9)
C32—C33—C34—C29	−3.5 (17)	N1A—S1A—C1A—F3A	56.0 (10)
C32—C33—C34—C35	174.3 (11)	N1A—S2A—C2A—F4A	54.2 (11)
C33—C34—C35—C36	−92.2 (13)	N1A—S2A—C2A—F5A	174.1 (8)
C33—C34—C35—C40	82.5 (13)	N1A—S2A—C2A—F6A	−69.2 (9)
C34—C29—C30—C31	−3.5 (15)	C1A—S1A—N1A—S2A	−103.8 (9)
C34—C35—C36—O4	−3.6 (16)	C2A—S2A—N1A—S1A	127.1 (8)
C34—C35—C36—C37	179.3 (12)		
